# Six-month prognostic impact of hemodynamic profiling by short minimally invasive monitoring after cardiac surgery

**DOI:** 10.34172/jcvtr.2020.62

**Published:** 2020-12-13

**Authors:** Cristina Giglioli, Emanuele Cecchi, Pier Luigi Stefàno, Valentina Spini, Giacomo Fortini, Marco Chiostri, Niccolò Marchionni, Salvatore Mario Romano

**Affiliations:** ^1^Division of General Cardiology, Department of Heart and Vessels, Azienda Ospedaliero-Universitaria Careggi, Florence, Italy; ^2^Division of Cardiosurgery, Department of Heart and Vessels, Azienda Ospedaliero-Universitaria Careggi, Florence, Italy; ^3^Department of Experimental and Clinical Medicine, Unit of Internal Medicine and Cardiology, University of Florence, Florence, Italy

**Keywords:** Cardiac-Surgery, Hemodynamic Profile, Clinical Six Months Follow-up

## Abstract

***Introduction:*** Studies have shown that a hemodynamic-guided therapy improves the post operative outcomes of high-risk patients.This study, evaluated if a short period through minimally invasive hemodynamic monitoring, pressure recording analytical method (PRAM), on admission to a post-cardiac surgery step-down unit (SDU), may identify patients at higher risk of 6-month adverse events after cardiac surgery.

***Methods:*** From December 2016-May 2017,173 patients were admitted in SDU within 24-48 hours of major cardiac surgery procedure, and submitted to clinical, laboratoristic and echocardiographic evaluation and a 1-hour PRAM recording to obtain a "biohumoral snapshot" of individual patient’s.156 173 patients (17 patients were lost at follow-up) were phone interviewed six months after surgery,to evaluate, as a composite end-point, the adverse events during follow-up. A multivariable logistic regression analysis was used to identify a model clinical-biohumoral (CBM) and clinical-biohumoral hemodynamics (CBHM).

***Results:*** No data from past clinical history and no conventional risk score (EuroScore II, STS score)independently predicted the risk of 6-month major events in our study. The risk of adverse events at six-month follow-up was directly related, in the CBM, to sustained post-operative cardiac arrhythmias, higher values of NT-proBNP and of arterial pH; inversely related to values of hs-C-reactive protein (hs-CRP) and, in the CBHM, to low values of cardiac cycle efficiency (CCE) and dP/dt_max_.

***Conclusion:*** Our study although limited by its observational nature and by the limited number of patients enrolled, showed that a short period of minimally invasive hemodynamic monitoring increased the accuracy to identify patients at major risk of mid-term events after cardiac surgery.

## Introduction

Based on multivariable risk models, several instruments (EuroScore I-II, Society of Thoracic Surgeons (STS score)) have been introduced into the clinical practice for global risk stratification in elective cardiac surgery, with the aim of facilitating patients’ selection, as well as timing and choice of surgical strategy.^1-5^

On the other hand, few tools are available for assessing the clinical stability and, hence, the appropriate patients’ stepping-down during the early course after surgery, especially in high-volume, high-turnover centers, while studies have shown that a hemodynamic-guided therapy improves the postoperative outcomes of high-risk patients.^6-11^ Pulmonary artery catheterization has been used for this purpose in the past, but the invasiveness of the method with its potentially serious complications,^12^ progressively has reduced its routine use. However, non-invasive, or minimally invasive, technologies are nowadays available for hemodynamic monitoring, which can be implemented even outside an intensive care setting. Among these, the pressure recording analytical method (PRAM) provides a reliable measure of cardiac output (CO), and of several derived parameters, only by means of a mathematical analysis of the pressure waveform recorded from a radial artery cannula.^13,14^ PRAM is a system that simultaneously analyses the systolic and diastolic phase and their interaction.^13^

The system analyses not only the area under the pressure curve above diastolic pressure, but it considers the entire area over zero pressure, taking in consideration not only the pulse pressure value but the whole pressure value: pulsatory and continuous contribution. The fundamental characteristic of this system is the analytical resolution, and the introduction of variables obtained during the whole cardiac cycle, without pre-calibrations and/or pre-estimated data coming from other patients. Data are obtained directly from to the investigated patient by the P/t (pressure/time) of each point of the whole cardiac cycle.^13,14^

In this study, we hypothesized that a short period of PRAM monitoring on admission to a post-cardiac surgery step-down unit (SDU), may independently contribute to identifying patients at higher risk of 6-month adverse events after cardiac surgery.

## Materials and Methods

All patients transferred to the SDU from the post-surgical intensive cardiac unit (PSICU) were considered eligible, provided they had undergone major cardiac surgery (coronary artery bypass surgery, cardiac valve replacement or combined intervention), they still had a radial artery cannula in place, and gave their informed consent. The patients submitted to cardiac surgery in emergency or in bail-out were excluded.

On admission to SDU, a complete clinical evaluation, arterial blood gas analysis, a standardized panel of laboratory tests including creatinine, troponin-I, NT-proBNP, procalcitonin, high-sensitivity C-reactive protein (hs-CRP), an electrocardiogram (ECG), and a 2-D and Doppler echocardiogram, were recorded. A 1-hour PRAM recording was taken to represent a “snapshot” of individual patient’s hemodynamic profile.

### Hemodynamic monitoring with PRAM

As previously described,^13,14^ from beat-to-beat analysis of radial artery pressure waveform, PRAM (MostCare-UP^®^, Vygon, Padua, Italy) provides continuous monitoring of CO, cardiac index (CI), stroke volume (SV), stroke volume variation (SVV), pulse pressure variation (PPV), systolic (SAP) and mean arterial pressure (MAP), dicrotic notch pressure (DP), dP/dt_max_, as well as of cardiac cycle efficiency (CCE) and arterial elastance (Ea), which assess ventricular-arterial coupling.^15-17^ Data are sampled at 1000 Hz, and averaged over each 30-sec period, resulting in 120 series of numeric values for one hour. The system requires neither calibration nor the introduction of patient’s data such as age or gender as variables are not derived on the basis of pre-estimated parameters; only few anthropometric data are required (height and weight) to obtain indexed values of hemodynamic parameters.

Many previous studies validated the PRAM hemodynamic parameters with that obtained by means of the thermodilution bolus, the direct Fick and the Ecocardiography.^13,17-21^

Because PRAM provide hemodynamic data from the arterial waveform analysis, a correct position of the arterial transducer with a proper “dumping” is crucial. Therefore, during the recording, the morphology of arterial wave was continuously checked by the physician in order to detect over or under-damping phenomenon and to optimize arterial waveform.^22-24^

### Follow-up and end-point definition

Six months after surgery, patients (or their relatives, in case of death) were phone interviewed by a physician who was unaware of the data recorded during hospital stay. The interview included the occurrence of hospital re-admissions, stroke/transient ischemic attack (TIA) ,^25^ worsening New York Heart Association (NYHA) functional class, or death, all included in a composite study end-point.

### Statistical analysis

Statistical analysis was performed with SPSS 20.0 package (IBM-SPSS, Chicago, USA). Continuous and categorical variables were reported respectively as mean ± standard deviation (or as median with 25th-75th percentile when non-normally distributed) and as frequencies and percentages. The Student *t* test or the Mann-Whitney *U* test, and the χ^2^ test were used to compare differences between study groups for continuous and categorical variables, respectively. The clinical, laboratory, ECG and echocardiographic parameters that, at univariable analysis, were significantly different between patients with and those without the 6-month composite end-point, were candidates’ variables to be entered into multivariable regression logistic models. A first, multivariable logistic regression analysis with backward selection method (*P* in <0.05, *P* out <0.10) was used to identify the clinical, laboratory, ECG and echocardiographic predictors of the composite end-point. The resulting model was forced into a second logistic regression analysis that contained the PRAM-derived hemodynamic variables significantly associated with events at follow-up, in order to obtain a final model with a backward selection algorithm. These two logistic models were verified by analysis of receiver operating characteristic (ROC) curves, and areas under the curves (AUC) were compared using the method described by Hanley and McNeil.^26^ All statistical tests were two-sided unless otherwise specified, and a *P* value <0.05 was considered statistically significant.

## Results

From December 2016, to May 2017, 173 patients were admitted to our SDU within 24 (64.7%) and 48 hours (82.1%) of major cardiac surgery procedure at Careggi Hospital, Florence, Italy, agreed to participate in the study and were discharged from hospital. Of these, 17 were lost to follow-up, while 156 (101 males; 64.7%; mean age 70.4 ± 10.3 years) were successfully traced 6 months after discharge.

**Figure 1:**
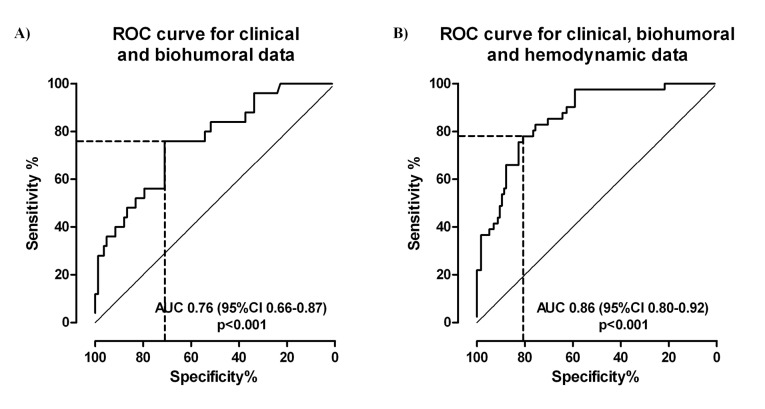
The ROC curves analysis to identify patients with adverse events at follow-up by **A**) standard multi-parametric evaluation and **B**) standard multi-parametric evaluation and hemodynamic data

No difference was found for any clinical, laboratory or instrumental variable between patients successfully traced and those lost to follow-up. Of 156 traced patients, 115 (73.7%) were event-free and 41 (26.3%) reported some adverse event (Table 1). In particular, 12 had died (5 of cardiovascular causes, 4 of respiratory problems and 3 of infections), and 29 had had a hospital re-admission (18 for cardiovascular and 11 for non-cardiovascular reasons). Compared to event-free patients, those with events had a higher prevalence of chronic kidney disease (CKD), and higher EuroScore II and Society of Thoracic Surgeons (STS) score. Relevant clinical data during SDU stay, and laboratory, echocardiographic and hemodynamic data on admission by 6-month events are reported in Table 2. Patients with events more frequently had had sustained cardiac arrhythmias (atrial fibrillation in almost all cases) during SDU stay, a slightly but significantly higher arterial pH, a lower hs-CRP, and a remarkably higher NT-proBNP on SDU admission. No echocardiographic parameter differed significantly between the two groups. Interestingly, 1-hour PRAM monitoring found that patients with 6-month events had lower cardiac efficiency and left ventricular contractility, a slightly but not significantly higher ventriculo-arterial coupling, and higher SVV and PPV, respectively suggesting a less efficient ventricle (CCE; dP/dt_max_; Ea) and a more unstable hemodynamic profile (Table 2). A first regression logistic model (Table 3, A) including sustained arrhythmias, CRP, NT-proBNP and arterial pH, had a 0.76 AUC (Figure 1 A), with 78% sensitivity and 71% specificity in predicting 6-month events. Of note, neither EuroScore II nor STS score were retained in this model as independent predictors of 6-month events. A second logistic regression model (Table 3 B) was then calculated with inclusion of hemodynamic variables that, at univariate analysis, were associated with 6-month events: of these, only CCE and dP/dt_max_ were retained in this second model. A final model was then calculated combing clinical and hemodynamic variables. This model had a good calibration (Hosmer-Lemeshow χ^2^ 13.14, *P* = 0.107) and showed a 0.86 AUC (Figure 1 B) with a similar sensitivity (79%) but a greater specificity (81%) than model A. Moreover, when comparing the two ROC curves (Figure 1) with the method described by Hanley and McNeil, the difference was found statistically significant (*P*= 0.016, one-tailed).^25^ Among clinical variables only NT-proBNP e arterial Ph were retained in this second model.

**Table 1:** Demographic and history data, surgical risk stratification, and type of cardiac surgery, in 156 patients with and without at 6-month events.

	**All patients** **n = 156**	**No events** **n = 115**	**Events** **n = 41**	***P*** ** value**
Age, mean±SD, years	70.4 ± 10.3	69.7 ± 10.5	71.3 + 9.5	0.390
Gender (M), n (%)	101 (64.7)	76 (66.1)	25 (61.0)	0.556
**History**				
Family history, n (%)	31 (19.9)	23 (20.2)	8 (19.5)	0.927
Ever smoker, n (%)	41 (26.3)	32 (27.8)	9 (22.0)	0.463
Arterial hypertension, n (%)	122 (78.2)	93 (80.9)	29 (70.7)	0.177
Chronic renal disease, n (%)	30 (19.2)	16 (13.9)	14 (34.2)	0.004*
COPD, n (%)	17 (10.9)	12 (10.4)	5 (12.5)	0.719
Diabetes, n (%)	39 (25.0)	25 (21.7)	14 (34.1)	0.172
Dyslipidemia, n (%)	72 (46.2)	53 (46.1)	19 (46.3)	0.978
Cerebrovascular events, n (%)	16 (10.3)	13 (11.3)	3 (7.5)	0.496
Malignancy, n (%)	11 (7.1)	7 (8.3)	4 (12.1)	0.528
NYHA class, n (%)				0.173
I	25 (16.0)	21 (18.3)	4 (9.8)	0.305
II	46 (29.5)	36 (31.3)	10 (24.4)	0.003*
III	68 (43.6)	49 (42.6)	19 (46.3)	0.818
IV	17 (10.9)	9 (7.8)	8 (19.5)	0.077
POAD, n (%)	29 (18.6)	18 (15.7)	11 (27.5)	0.098
Previous AMI, n (%)	38 (24.4)	24 (20.9)	14 (35.0)	0.074
Previous CABG, n (%)	9 (5.8)	5 (4.3)	4 (9.8)	0.202
Previous PCI, n (%)	28 (17.9)	18 (15.7)	10 (24.4)	0.211
**Cardiac Surgery Risk Stratification**				
EuroSCORE II, median (IQR)	6.2 (2.6 - 11.7)	5.24 (2.3 - 10.4)	8.09 (4.59 - 20.52)	0.002*
STS score, median (IQR)	3.1(1.8 - 5.0)	2.8 (1.4 - 4.6)	4.8 (2.6 - 8.9)	0.004*
**Type Cardiac Surgery**				0.143
Isolated CABG- Other surgery	40 (25.7)	31 (27.0)	9 (21.9)	
Surgery on single-multi valves	94 (60.2)	67 (62.1)	24 (58.5)	
Combined CABG + valves	22 (14.1)	14 (12.2)	8 (19.5)	

Abbreviations: COPD, chronic obstructive pulmonary disease; NYHA, New York Heart Association; POAD, peripheral obstructive artery disease; AMI, acute myocardial infarction; CABG, coronary artery by-pass graft; PCI, percutaneous coronary intervention; EuroSCORE, European System for Cardiac Operative Risk Evaluation; STS, Society of Thoracic Surgeons;IQR,interquartile range *Statistically significant

**Table 2:** Sustained cardiac arrhythmias and laboratory, echocardiographic and hemodynamic (from PRAM 1-hour monitoring) data for 156 patients with and without events at 6-month follow-up.

	**No events** **n=115**	**Events** **n=41**	***P*** ** value**
**Cardiac Arrhythmias**	41 (35.7)	26 (63.4)	0.004*
**Laboratory**			
Hb nadir, g/dL	9.8 +1.6	9.2 + 1.4	0.058
Peak TnI, ng/mL, median (IQR)	2.54 (0.77 - 5.46)	1.92 (0.94 - 4.73)	0.559^a^
eGFR(CKD-EPI), mL/min/1.73m^2^ ,median (IQR)	72.5 (50.2 - 104.1)	62.4 (32.3-105.3)	0.093^a^
Arterial pH (units)	7.45 + 0.04	7.47 + 0.05	<0.023
Arterial lactate, mmol/L	1.2 + 0.7	1.3 + 0.5	0.419
Arterial PaO_2_, mm Hg	114 + 85	106 + 45	0.561
Arterial PaCO_2_, mm Hg	41 + 4	40 + 5	0.368
CRP, median (IQR)	143 (116 - 172)	102 (69 - 143)	<0.001^a^
NT-proBNP, pg/mL, median (IQR)	3453 (1895 - 6565)	5161 (3045 - 19325)	0.004^a^
**Echocardiography**			
LAA, cm^2^	26.0 + 8.5	26.5 + 9.0	0.731
IVS, mm	11.8 + 1.9	11.4 + 1.5	0.207
PW, mm	11.0 + 1.4	11.1 + 1.3	0.532
EDV, mL	100 ± 45	113 ± 54	0.119
LVEF (%)	51.6 + 9.6	47.4 + 11.8	0.026
TAPSE, mm	16.3 + 3.5	16.1 + 4.4	0.804
**Hemodynamics**			
PR, bpm	78 ± 8	79 ± 10	0.505
Systolic/Diastolic arterial pressure, mm Hg	138/63 ± 19/11	138/62 ± 17/9	0,112/0.600
MAP, mm Hg	88 ± 12	86 ± 11	0.308
CI, L/min/m^2^ ; median (IQR)	2.44 (2.19 - 2.50)	2.27 (2.01 - 2.58)	0.574 ^a^
Systemic vascular resistance, dyne*s*cm-^5^	1473 ± 206	1479 ± 223	0.886
CCE (units)	0.15 ± 0.17	-0.03 ± 0.29	<0.001^*^
dP/dt_max_ , mm Hg/msec	1.16 ± 0.26	1.00 ± 0.24	<0.001^*^
SVV (%)	15.5 ± 5.8	20.7 ± 10.2	<0.001^*^
PPV(%)	22.2 ± 10.9	30.9 ± 16.7	<0.001^*^
Ea, mm Hg/mL	1.4 ± 0.3	1.5 ± 0.4	0.120

Abbreviations: Hb, hemoglobin; TnI, troponin I; eGFR, estimated glomerular filtration rate; CKD, cronic kidney diseases CRP, C-reactive protein; NT-proBNP, N-Terminal pro Brain Natriuretic Peptide; LAA, left atrial area; IVS, Interventricular septum; PW, posterior wall; EDV, end diastolic volume ; LVEF, left ventricle ejection fraction; TAPSE, tricuspid annular plane systolic excursion; PR, pulse rate; MAP, mean arterial pressure; CI, cardiac index; CCE, cardiac cycle efficiency; dP/dt_max_, Peripheral artery dP/dt_max_; SVV, stroke volume variation; PPV, pulse pressure variation; Ea, arterial elastance; IQR,interquartile range Values are mean±SD if not otherwise specified. All comparisons are computed with Student *t* test but (a) Mann-Whitney *U* test

**Table 3:** The clinical-biohumoral model A) plus hemodynamics model variables B) on 6-month prognosis

	**Adjusted OR**	**95% CI**	***P*** ** value**
**A) Clinical & biohumoral model**			
Cardiac arrhythmias	2.368	1.053 –5.322	0.037
CRP (1unit step)	0.991	0.985 – 0.998	0.007
NT-proBNP (1000unit step)	1.006	1.001 – 1.011	0.018
Arterial pH (0.01 units step)	1.109	1.001 – 1.230	0.048
**B) Clinical & biohumoral and hemodynamic model**			
Cardiac arrhythmias	2.059	0.837 –5.068	0.116
CRP (1unit step)	0.994	0.987 – 1.001	0.114
NT-proBNP (1000 unit step)	1.006	1.001 – 1.012	0.031
Arterial pH (0.01 units step)	1.134	1.00 – 1.261	0.047
CCE (1 unit step)	0.016	0.001 – 0.176	<0.001
dP/dt_max_ (1 mm Hg/msec step)	0.157	0.023 – 1.070	0.059

Abbreviations: CRP, C-reactive protein; NT-proBNP, N-Terminal pro Brain Natriuretic Peptid**e**; CCE, cardiac cycle efficiency; dP/dt_max_, Peripheral artery dP/dt_max_. ;OR,odds ratio; CI,confidence interval Hosmer-Lemeshow goodness-of-fit χ^2^ 5.16, *P* = 0.741; Nagelkerke pseudo R^2^ 0.28. This model has a correct classification rate (efficiency) 0.78 (95% CI 0.71-0.84); sensitivity 0.32 (95% CI 0.18 - 0.48); specificity 0.95 (95% CI 0.89 - 0.98). Predictive value of positive test 0.68 (95% CI 0.43 - 0.87); predictive value of negative test 0.80 (95% CI 0.72 - 0.86). Hosmer-Lemeshow goodness-of-fit χ^2^ 13.14, *P* = 0.107; Nagelkerke pseudo R^2^ 0.45. This model has a correct classification rate (efficiency) 0.79 (95% CI 0.72 - 0.85); sensitivity 0.41 (95% CI 0.26 - 0.58); specificity 0.92 (95% CI 0.86 - 0.96). Predictive value of positive test 0.65 (95% CI 0.44 - 0.83); predictive value of negative test 0.82 (95% CI 0.74 - 0.88).

## Discussion

The major findings of our study were that, in cardiac surgery patients, no data from past clinical history and no conventional risk score (EuroScore II, STS score) independently predicted the risk of 6-month major events. The risk of events was in fact directly related to the occurrence of sustained post-operative cardiac arrhythmias, higher values of NT-proBNP and of arterial PH, and inversely related to values of CRP. Moreover, it is interesting to note that adding a minimally invasive, short duration (one hour) PRAM monitoring, significantly improved the predictive efficiency of a multidimensional regression logistic model, with low values of CCE and dP/dt independently associated with higher risk of 6-month events.

Some of these findings deserve detailed comments. In keeping with their reported indication for clinical use, both Euroscore II and STS were higher in the group with 6-month events, but only at univariate analysis. However, since in multivariable models they did not result to be independent predictors of 6-month prognosis, this confirmed their limited utility, in our study population, in risk stratification beyond the immediate post-operative period.^27-29^ In accordance with previous findings,^29-32^ CKD was more prevalent in patients with than in those without 6-month events, though glomerular filtration rate estimated (eGFR) from CKD-EPI formula on admission to SDU was only marginally lower in patients with 6-month events, as a possible consequence of the hyper-hydration that is a standard of the immediate post-operative period. However, neither eGFR nor a history of CKD were independent predictors of events, probably because of the longer observation period needed for CKD to emerge as a risk predictor, particularly when simultaneously adjusting for other clinical or pathophysiological variables that are more powerful predictors short-term. Among laboratory data, hemoglobin values were lower in patients with 6-month events. Anemia is a clinically important and increasingly frequent finding in patients presenting for surgery. An association of anemia with increased perioperative morbidity and mortality has been established in a multitude of settings including cardiac surgery.^33,34^ In particular, the negative prognostic impact in CABG patients has been attributed to anemia-induced myocardial ischemia in the context of limited coronary reserve.^35^ However, this does not seem relevant in our series, as troponin I values were similar in the two groups, thereby rejecting the hypothesis of greater myocardial damage as a consequence of more marked anemia in patients with subsequent events. In previous studies, elevated pre-operative NT-proBNP was found a potent predictor of negative events from 1^36^ to 5 years^37^ after cardiac surgery: we found that a predictive effect is already evident at 6 months, thereby confirming the usefulness of assaying this parameter also in the early post-operative days. In relation to the arterial PH value it is important to underline that the difference among 7.45 and 7.47, observed in patients without and with events at follow-up, although statistical significant it is not clinically remarkable. Moreover, in our study population the risk of events was also related to the occurrence of sustained post-operative cardiac arrhythmias (atrial fibrillation in almost all cases). Atrial fibrillation is the most common arrhythmia in patients submitted to cardiac surgery. Several mechanisms are involved in the genesis of this arrythmia^38^ of electrical and mechanical nature Generally atrial fibrillation, in this group of patients, is paroxysmal of brief duration and responsive to amjodarone infusion. If the rate is not very high and the cardiac function not very low, AF is not a relevant clinical event. In our study that is a real world study 67 patients (38%) had AF during hospital stay but these events did not invalidate our results for the following reasons: 1) Only 10 patients, during PRAM monitoring, were in persistent atrial fibrillation with a mean ventricular rate 90b/min. We previously published a work about the hemodynamic advantages, evaluated by means of PRAM, of the electric cardioversion in patients with persistent AF where we demonstrated the clinical validity of PRAM (if the waveform is appropriate) to calculate hemodynamic parameters also with this arrhythmia^39^. 2) The other patients were in sinus rhythm, PRAM monitoring started at the SDU admission, but at our institution the passage from the IC to SDU is possible only if the patient is clinically e and hemodynamically stable. 3) In our final regression model including clinical and hemodynamic variables AF did not result significant and was excluded.

Interestingly the hemodynamic “snapshot” obtained with 1-hour PRAM monitoring showed significantly lower post-operative CCE in patients with compared to these without 6-month adverse events, while CI and SV were similar in the two groups. CCE describes the cardiovascular performance in terms of ratio between hemodynamic work performed and energy expenditure,^15,17^ thereby assessing the ability of the cardiovascular system to maintain homeostasis at different energy levels. This result suggests that the 41 patients who developed 6-month adverse events had a hemodynamic profile characterized by greater energy expenditure to maintain CI and SV values similar to those of the 115 patients without events. Patients with adverse events also had lower values of dp/dt_max_ and higher values of Ea, suggesting respectively lower left ventricular contractility and higher vascular load.^17^ Moreover, larger SVV and PPV indicating greater hemodynamic instability in patients with 6-month events, are in accordance with the concept that hemodynamically unstable patients have difficulties in reaching an adequate physiological equilibrium. In accordance with these findings and pathophysiological interpretation, the logistic regression and the ROC curve analysis showed that hemodynamic profiling with PRAM significantly improved the precision of a more conventional, even though multi-parametric, evaluation, in identifying patients at increased risk of adverse events at follow-up.

This is a mono-centric, observational study that included a limited number of patients. Moreover, other limitation of this study is that our model has not been externally validated with a separate set of patients. This first algorithm retrospectively predicted the final effect of the Clinical-biohumoral and hemodynamic model within the cohort of the patients. However, it is a real word-study that well reflect our daily clinical practice.

## Conclusion

The adoption of multiple systems to assess the risk of adverse events at mid-term follow-up would greatly improve risk stratification and, hence, clinical decision making after cardiac surgery patients. Each system has limitations that probably can be overcome by enriching the assessment methods with accurate pathophysiological information. In particular, though our study is limited by its observational nature and the somewhat limited number of patients enrolled, we are convinced that an even short period of minimally invasive hemodynamic monitoring represents a clinically valuable, minimally invasive and low-cost method to improve the accuracy in identifying patients at increased risk of mid-term events after cardiac surgery.

## Competing interest

The authors declared the following potential conflicts of interest with respect to the research, authorship, and/or publication of this article: Dr Salvatore Mario Romano is owner of the patent for continuous cardiac output method; no conflict for the other authors.

## Ethical approval

This study was a non-profit, single center, observational and each patient gave their written consent before each procedure to authorize anonymously the collected data, in agreement with the previous protocol approval by the local ethical committee (Azienda Ospedaliero-Universitaria Careggi, Firenze, Area Vasta Centro, Italia; ref. number CE OSS 15144).

## Funding

None.
